# Drug-Related Problems in HIV Treatment Failure

**DOI:** 10.7759/cureus.69838

**Published:** 2024-09-21

**Authors:** Mohd Farizh Che Pa, Ng Tiang Koi, Arisah Misnan, Farida Hanim Islahudin, Mohd Makmor-Bakry

**Affiliations:** 1 Center of Quality Medicine Management, Faculty of Pharmacy, Universiti Kebangsaan Malaysia, Kuala Lumpur, MYS; 2 Pharmacy, Hospital Tengku Ampuan Rahimah, Klang, MYS; 3 Internal Medicine, Hospital Tuanku Ja'afar, Seremban, MYS; 4 Internal Medicine, Hospital Sungai Buloh, Sungai Buloh, MYS

**Keywords:** antiretroviral therapy, drug-related problems, hiv, pharmaceutical care, treatment failure

## Abstract

Background

Antiretroviral therapy (ART) is used in human immunodeficiency virus (HIV) treatment to reduce morbidity and mortality rates among people living with the virus. The types of drug-related problems (DRPs) and their causes that contributed to HIV treatment failure were unknown. Thus, this study aimed to determine the types and causes of DRPs associated with HIV treatment failure.

Methods

A multicentre, retrospective cohort study was conducted at the Infectious Disease Centre of Sungai Buloh Hospital, Selangor, and Tuanku Ja’afar Hospital, Negeri Sembilan. Data were collected from patients’ medical records by reviewing the medical progress notes, laboratory parameters, and treatment regimen. Pharmaceutical Care Network of Europe’s (PCNE) classification system V9.1 was used to identify and classify the types and causes of DRPs. Patients were classified as having treatment failure if the HIV RNA viral load was more than 1000 copies/ml for two consecutive readings. Patients were categorized as treatment successful if there was a decrease in HIV RNA viral load suppression after six months of ART initiation and had no history of persistent HIV RNA viral load greater than 1000 copies/ml for two consecutive measurements. Data were analyzed using the chi-square test.

Results

The number of patients recruited for this study was 355 (treatment success group = 263, treatment failure group = 92). Almost all patients (n = 354, 99.7%) had experienced at least one DRP. A total of 811 problems and 1605 causes of DRPs were encountered. The occurrence of DRPs in the HIV treatment failure group was 5.77 DRPs per patient compared to the success group (4.08 DRPs per patient). In the treatment failure group, treatment effectiveness was identified as the most frequent domain of problems (P) (P1: n = 169, 59.93%), followed by treatment safety (P2: n = 111, 39.36%). The common domains of causes (C) include patient-related (C7: n = 367, 69.11%), other (C9: n = 105, 19.77%), and drug selection (C1: n = 49, 9.23%). Significant differences were found in several causes (C7.1, C7.10, and C9.1) between the two groups.

Conclusion

The occurrence of DRPs in HIV treatment failure was high, contributing to treatment effectiveness and treatment safety issues.

## Introduction

Globally, up to 2022, there were 39.0 million people living with human immunodeficiency virus (PLHIV), and 89% of them started on antiretroviral therapy (ART) [1]. In 2021, the estimated number of PLHIV in Malaysia was 81,942, and 66% of them received ART [2]. The treatment of HIV has evolved significantly over the years. After the approval of zidovudine as the first antiretroviral drug (ARV) in 1987, the use of monotherapy or single-drug regimens was common, but this often led to drug resistance and treatment failure [3].

The combination of ARV, often referred to as ART, became the standard treatment started in 1995, soon after lamivudine approval [4]. The use of protease inhibitors, non-nucleoside reverse transcriptase inhibitors, and nucleoside reverse transcriptase inhibitors in various combinations of therapy became more widespread [5]. 

PLHIV were encouraged to start ART as early as possible to significantly reduce HIV viral load, improve immune function, and prolong survival [6,7]. The introduction of ART was estimated to prevent 9.5 million HIV-related deaths and 7.9 million HIV new infections between 1995 and 2015 [8]. A study conducted among hospitalized patients with HIV infection demonstrated that 67-86% of the patients had at least one drug-related problem (DRP) related to ART [9,10]. A five-year followed-up retrospective study showed the occurrence of adverse drug reactions (ADRs) on ART was 24.73% [11]. A study in outpatient settings showed the occurrence of 5.0 DRPs per patient [12]. Nevertheless, the occurrence of DRPs that are attributed to HIV treatment failure is uncertain.

The Pharmaceutical Care Network Europe (PCNE) classification system is the most extensively used in clinical practice to classify any DRPs [13]. The classification is periodically updated and revised to ensure internal consistency. Prior research suggested that pharmacists could utilize the PCNE classification system to document DRPs in accordance with a standardized pattern [14,15]. Hence, this study aimed to identify the types and causes of DRP associated with HIV treatment failure based on PCNE classification.

## Materials and methods

Study setting and design

This retrospective cohort study was conducted as a multicenter at two main infectious disease referral centers for Malaysia, namely, Sungai Buloh Hospital, Selangor, and Tuanku Ja’afar Hospital, Negeri Sembilan. The study inclusion criteria were HIV patients aged more than 18 years and started on ART from 2010 to 2020 for at least six months before the first follow-up visit. Patients with incomplete data and transferred from other facilities were excluded. Patients were defined as having treatment failure if the HIV RNA viral load showed more than 1000 copies/ml for two consecutive readings at clinic visits. Patients were categorized as treatment successful if there was a decrease in HIV RNA viral load suppression after six months of ART initiation and had no history of persistent HIV RNA viral load more than 1000 copies/ml for two consecutive measurements. The sample size was calculated using the formula suggested by Peduzzi et al. 1996 for association analysis [16]. The sample size was estimated at 122 based on the assumption of two significant domains of DRP classification [12], prevalence of HIV treatment failure at 0.18% [17], and consideration of 10% of missing data.

Study protocol

All patients who were diagnosed with HIV from each center were listed as potential candidates. The list was updated based on inclusion and exclusion criteria. From the patient list, subjects were selected by simple random sampling. Patient characteristics, medical progress notes, drug prescribed, and laboratory results were extracted from the medical records. Drug-related variables that were recorded include the type of ART prescribed, DRPs identified, inappropriate drug combination with other drugs, herbal or dietary supplements, adherence status, ADR, and therapy monitoring.

DRPs identified were categorized using the PCNE classification V9.1 [18]. The basic classification of PCNE includes problems (P), causes (C), interventions (I), acceptance of interventions (A), and status of DRPs after the intervention (O) [18]. For the purpose of this study, only problems (P) and causes (C) of each DRP were focused. The causality assessment of each DRP was independently classified by one clinical pharmacist with at least five years of experience in HIV management. The causality criteria were based on any possible causes associated with the problems. The identification of DRPs involves a comparison of drug dosage and frequency, possible drug interaction, method of administration between the prescribed ART, and information in established references. The references include the Malaysian Consensus Guideline on Antiretroviral Therapy 2022 [19] and Guidelines for the Use of Antiretroviral Agents in Adults and Adolescents with HIV 2020 [20]. Additional references include electronic databases, Micromedex [21], and HIV drug interactions by the University of Liverpool [22]. A consensus discussion between co-investigators (AM and NTK) was conducted to confirm uncertainties on the classification of the identified DRPs. Unresolved problems and final confirmation of the DRPs were further discussed with additional co-investigators (FI and MMB)

Statistical analysis

The patients were categorized into two groups, treatment failure and treatment success. Mean with standard deviation (SD) and frequency were used to describe the patient characteristics. For DRP comparison between groups, a chi-square test was used to derive the p-value. For all comparisons, p less than 0.05 was considered statistically significant. All the data were analyzed using IBM SPSS Statistics for Windows, Version 27 (Released 2020; IBM Corp., Armonk, New York, United States).

Ethical consideration

Consent to review patient medical records was obtained prior to data collection. The Malaysian Medical Research Ethical Committee (NMRR ID-22-02618-MAB (IIR)) and Universiti Kebangsaan Malaysia Ethical Committee (UKM PPl/111/8/JEP-2023-115) approved this study. The study complied with the Declaration of Helsinki and patient confidentiality.

## Results

Demographic characteristics

A total of 355 patients were enrolled in this study. Of that, 92 patients (25.9%) were categorized as treatment failure, and 263 patients (74.1%) were categorized as treatment success. Overall, a majority of patients were Malay (n = 196, 55.2%), single (n = 187, 52.7%), and non-professional (n = 210, 59.2%). The most common mode of transmission was heterosexual (n = 147, 41.%). The mean duration of ART was 7.72 ± 4.11 years. The majority of the patients had co-morbidities (n = 186, 52.4%) and initiated ART with a non-nucleoside reverse transcriptase inhibitor-(NNRTIs) based regimen (n = 349, 98.3%). The demographic characteristics of the study population with drug-related problems are summarized in Table 1. There were no statistically significant differences observed in age, gender, race, occupation, marital status, alcohol consumption, co-morbidities, or concurrent medications between patients in both groups.

**Table 1:** Characteristics of study participants based on the presence of HIV treatment failure ^a^Independent T-test; ^b^Pearson chi-square test IVDU: intravenous drug users; ART: antiretroviral therapy; NNRTI: non-nucleoside reverse transcriptase inhibitors; INSTI: integrase strand transfer inhibitors

Characteristics	Overall, n=355	Treatment Status		P-value
		Failure, n=92 (25.9%)	Success, n=263 (74.1%)	
Age, mean (SD)	43.08 (±10.70)	43.85 (±10.38)	42.81 (±10.82)	0.426^a^
Gender				0.211^b^
Male	311 (87.6)	84 (91.3)	227 (86.3)	
Female	44 (12.4)	8 (8.7)	36 (13.7)	
Race				0.994^b^
Malay	196 (55.2)	51 (55.4)	145 (55.1)	
Chinese	98 (27.6)	26 (28.3)	72 (27.4)	
Indian	45 (12.7)	11 (12.0)	34 (12.9)	
Others	16 (4.5)	4 (4.3)	12 (4.6)	
Occupation				0.103^b^
Unemployed	57 (16.0)	21 (22.8)	36 (13.7)	
Professional	88 (24.8)	19 (20.7)	69 (26.2)	
Non-professional	210 (59.2)	52 (56.5)	158 (60.1)	
Marital Status				0.110^b^
Single	187 (52.7)	56 (60.9)	131 (49.8)	
Married	99 (27.9)	18 (19.6)	81 (30.8)	
Divorce/Widowed	34 (9.6)	11 (12.0)	23 (8.7)	
In relationship	35 (9.8)	7 (7.6)	28 (10.6)	
Smoking Status				0.030^b^
Yes	118 (33.2)	39 (42.4)	79 (30.0)	
No	237 (66.8)	53 (57.6)	184 (70.0)	
Alcohol Consumption				0.656^b^
Yes	64 (18.0)	18 (19.6)	46 (17.5)	
No	291 (82.0)	74 (80.4)	217(82.5)	
Mode of Transmission				<0.001^b^
Unknown	38 (10.7)	5 (5.4)	33 (12.5)	
Homosexual	144 (40.6)	35 (38.0)	109 (41.4)	
Heterosexual	147 (41.4)	34 (37.0)	113 (43.0)	
IVDU	26 (7.3)	18 (19.6)	2 (6.7)	
Duration on ART (years)	7.72 (±4.11)	9.70(±4.07)	7.03(±3.90)	<0.001^a^
Comorbidities				0.593^b^
Yes	186 (52.4)	46 (50.0)	140 (53.2)	
No	169 (47.6)	46 (50.0)	123 (46.8)	
Concurrent Medication				0.997^b^
Yes	162 (45.6)	42 (45.7)	120 (45.6)	
No	193 (54.4)	50 (54.3)	143 (54.4)	
Baseline CD4				0.020^b^
<200	206 (58.0)	66 (71.7)	140 (53.2)	
>200	149 (42.0)	26 (28.3)	123 (46.8)	
Initial Regimen				NA
NNRTI based	349 (98.3)	92 (100.0)	257 (97.7)	
PI based	2 (0.6)	0 (0.0)	2 (0.8)	
INSTI based	4 (1.1)	0 (0.0)	4 (1.5)	
Current Regimen				<0.001^b^
NNRTI based	218 (61.4)	0 (0.0)	217 (82.5)	
PI based	86 (24.2)	79 (85.9)	7 (2.7)	
INSTI based	51 (14.4)	13 (14.1)	39 (14.8)	
History of Regimen Changes				<0.001^b^
Yes	186 (52.4)	92 (100)	94 (35.7)	
No	169 (47.6)	0 (0.0)	169 (64.3)	

Types and causes of DRPs

Almost all patients (n = 354, 99.7%) had at least one DRP. Only one patient in the treatment success group did not experience any DRPs. A total of 817 problems and 1605 causes were encountered. The detailed distribution of the types of problems and causes is presented in Table 2.

**Table 2:** Details of problems and causes of DRPs identified in the study population DRPs: drug-related problems; TDM: therapeutic drug monitoring

Primary domain	Code	Nature of DRPs	Treatment Status				Total		P-value
			Failure n = 92, 25.9%		Success, n = 263, 74.1%		n = 355		
			No of DRPs (%)	No of DRPs per patient	No of DRPs (%)	No of DRPs per patient	No of DRPs (%)	No of DRPs per patient	Chi-square
P1: treatment effectiveness	P1	All P1 problems	169 (59.93%)	1.84	171 (31.96%)	0.65	340 (41.62%)	0.96	<0.001
	P1.1	No effect of drug treatment	0 (0%)		0 (0%)		0 (0%)		>0.95
	P1.2	Effect of drug treatment not optimal	169 (59.93%)		171 (31.96%)		340 (41.62%)		<0.001
	P1.3	Untreated symptoms or indication	0 (0%)		0 (0%)		0 (0%)		>0.95
P2: treatment safety	P2	All P2 problems	111 (39.36%)	1.21	364 (68.04%)	1.38	475 (58.14%)	1.34	0.398
	P2.1	Treatment safety	111 (39.36%)		364 (68.04%)		475 (58.14%)		0.398
P3: others	P3	All P3 problems	2 (0.71%)	0.02	0 (0%)	0	2 (0.20%)	0.01	0.117
	P3.1	Problem with cost-effectiveness of the treatment	0 (0%)		0 (0%)		0 (0%)		>0.95
	P3.2	Unnecessary drug treatment	2 (0.71%)		0 (0%)		2 (0.24%)		0.117
	P3.3	Unclear problem/complaint	0 (0%)		0 (0%)		0 (0%)		>0.95
		Total number of problems	282 (100%)	3.07	535 (100%)	2.03	817 (100%)	2.30	0.003
C1: drug selection	C1	All C1 causes	49 (9.23%)	0.53	92 (8.57%)	0.35	141 (8.79%)	0.40	0.050
	C1.1	Inappropriate drug according to guidelines/formulary	3 (0.56%)		7 (0.65%)		10 (0.62%)		0.938
	C1.2	Inappropriate drug (within guidelines but contraindicated)	3 (0.56%)		2 (0.19%)		5 (0.31%)		0.227
	C1.3	No indication for drug	0 (0%)		0 (0%)		0 (0%)		>0.95
	C1.4	Inappropriate combination of drugs, or drugs and herbal medications, or drugs and dietary supplements	41 (7.72%)		83 (7.73%)		124 (7.73%)		0.126
	C1.5	Inappropriate duplication of a therapeutic group or active ingredient	0 (0%)		0 (0%)		0 (0%)		>0.95
	C1.6	No or incomplete drug treatment in spite of existing indication	2 (0.38%)		0 (0%)		2 (0.12%)		0.117
	C1.7	Too many drugs prescribed for indication	0 (0%)		0 (0%)		0 (0%)		>0.95
C2: drug form	C2	All C2 causes	0 (0%)	0	3 (0.28%)	0.01	3 (0.19%)	0.01	0.719
	C2.1	Inappropriate drug form (for this patient)	0 (0%)		3 (0.28%)		3 (0.19%)		0.719
C3: dose selection	C3	All C3 causes	1 (0.19%)	0.01	3 (0.28%)	0.01	4 (0.25%)	0.01	0.595
	C3.1	Drug dose too low	1 (0.19%)		0 (0%)		1 (0.06%)		0.586
	C3.2	Drug dose too high	0 (0%)		0 (0%)		0 (0%)		>0.95
	C3.3	Dosage regimen not too frequent enough	0 (0%)		0 (0%)		0 (0%)		>0.95
	C3.4	Dosage regimen too frequent	0 (0%)		2 (0.19%)		2 (0.12%)		>0.95
	C3.5	Dose timing instructions wrong, unclear, or missing	0 (0%)		1 (0.09%)		1 (0.06%)		0.586
C5: dispensing	C5	All C5 causes	9 (1.69%)	0.10	12 (1.12%)	0.05	21 (1.31%)	0.06	0.088
	C5.1	Prescribed drugs not available	3 (0.58%)		5 (0.47%)		8 (0.50%)		0.741
	C5.2	Necessary information not provided	2 (0.38%		1 (0.09%)		3 (0.19%)		0.347
	C5.3	Wrong drug, strength, or dosage advised (OTC)	2 (0.38%)		4 (0.37%)		6 (0.37%)		>0.95
	C5.4	Wrong drug or strength dispensed	2(0.38%)		2 (0.19%)		4 (0.25%)		0.605
C7: patient-related	C7	All C7 causes	367 (69.11%)	3.99	582 (54.19%)	2.21	937 (58.38%)	2.64	<0.001
	C7.1	Patient uses/takes less drug than prescribed or does not take the drug at all	143 (26.93%)		177 (16.48%)		320 (19.94%)		<0.001
	C7.2	Patient uses/takes more than prescribed	0 (0%)		1 (0.09%)		1 (0.06%)		0.580
	C7.3	Patient abuses drug (unregulated overuse)	0 (0%)		0 (0%)		0 (0%)		>0.95
	C7.4	Patient takes a drug that interacts	26 (4.90%)		67 (6.24%)		93 (5.79%)		0.806
	C7.5	Patient takes food that interacts	17 (3.20%)		68 (6.33%)		85 (5.30%)		0.256
	C7.6	Patient stores drug inappropriately	1 (0.19%)		2 (0.19%)		3 (0.19%)		0.712
	C7.7	Inappropriate timing or dosing intervals	31 (5.84%)		95 (8.85%)		126 (7.85%)		0.772
	C7.8	Patient administers/uses the drug in a wrong way	0 (0%)		1 (0.09%)		1 (0.06%)		0.581
	C7.9	Patient unable to use drug/form as directed	0 (0%)		1 (0.09%)		1 (0.06%)		0.581
	C7.10	Patient unable to understand instructions properly	149 (28.06%		172 (16.10%)		321 (20.00%)		<0.001
C9: other	C9	All C9 causes	105 (19.77%)	1.14	382 (35.57%)	0.40	487 (30.34%)	1.37	0.140
	C9.1	No or inappropriate outcome monitoring (including TDM)	13 (2.45%)		128 (11.92%)		141 (8.79%)		<0.001
	C9.2	Other cause: Insufficient supply	2 (0.38%)		0 (0%)		94 (5.58%)		0.117
	C9.3	No obvious cause	90 (16.95%)		254 (23.65%)		344 (21.43%)		0.938
	Total number of causes (one DRP may have one or more causes)		531 (100%)	5.77	1074 (100%)	4.08	1605 (100%)	4.52	0.014

Overall, treatment safety was the most frequent problem domain identified (P2: n = 475, 58.14%; 1.34 DRPs per patient), followed by treatment effectiveness (P1: n = 340, 41.62%; 0.96 DRPs per patient). Focusing on the treatment failure group, the most frequent problem domain was treatment effectiveness (P1: n = 169, 59.93%; 1.84 DRPs per patient), followed by treatment safety (P2: n = 111, 39.36%; 1.21 DRPs per patient). While in the treatment success group, the most frequent problem domain identified was treatment safety (P2: n = 364, 68.04%; 1.38 DRPs per patient), followed by treatment effectiveness (P1: n = 171, 31.96%; 0.65 DRPs per patient). 

Among the seven domains of causes, the most common domain was patient-related (C7: n = 937, 58.38%; 2.64 DRPs per patient), followed by other (C9: n = 487, 30.34%; 1.37 DRPs per patient), and drug selection (C1: n = 141, 8.79%; 0.40 DRPs per patient). Types of causes were recognized in the treatment failure group and treatment success group, but some of the causes have significantly different percentages, namely C7.1 (patient uses/takes less drug than prescribed or does not take the drug at all), C7.10 (patient is unable to understand instructions properly), and C9.1 (no or inappropriate outcome monitoring, including TDM).

## Discussion

The present study showed, that in an outpatient setting, the frequency of DRPs was 4.52 per patient. This result was slightly lower compared to two prospective studies for similar settings, which reported 5.2 and 5.0 DRPs per patient [12,23]. However, these two studies specifically examined the impact of pharmacist interventions on HIV patients and employed different DRP classifications. These two studies included all interventions and were not restricted to ART only. Another factor might be due to the different DRP classifications used. Our study was specifically on ARV drugs only, which may influenced the number of DRPs observed. Prophylaxis therapy, treatment for opportunistic infections, and other drugs for comorbidities were not accounted for. In comparison to published data on hospitalized patients [10], our frequency of DRPs was higher. Outpatient settings have a higher tendency for DRPs, as strict follow-up and monitoring may be lacking [24]. 

Numerous studies were carried out using the same PCNE classification in various clinical conditions, which have reported a lower frequency of DRPs. A lower prevalence of DRPs was reported in heart failure (2.80 DRPs per patient) [25], stroke (0.32 DRPs per patient) [26], rheumatoid arthritis (1.6 DRPs per patient) [15], and neurology (0.25 DRPs per patient) [27]. This describes a high level of complexity in HIV management, which requires at least two ARVs from two different classes as an initial regimen along with close monitoring.

Focusing on the treatment failure group, the most common problem domain was treatment effectiveness. It was associated with the drug treatment not being optimal. Inappropriate combination of drugs, herbal and dietary supplements, drug-drug interactions, adherence level, inappropriate timing of drug taking, unable to understand instruction properly, and inappropriate monitoring contributed to the problem. In several cases, the potency of ARV medications remains unaffected but necessitates modification due to drug-drug interactions that impair the effectiveness of other medications. Coadministration of efavirenz with daclatasvir in a coinfected hepatitis C patient, as an example, requires a dose adjustment of daclatasvir [28]. Concomitant use of lopinavir/ritonavir with rifampicin is not recommended as it causes a large reduction in lopinavir concentration, which may in turn significantly reduce the lopinavir therapeutic effect and lead to HIV treatment failure [19].

The second most common problem domain of DRPs in this study was associated with treatment safety, which was ADRs. This result was consistent with previous studies conducted in similar settings [12,23,29]. ART typically involves a combination of several ARV drugs to effectively suppress HIV replication. This combination increases the potential for drug interactions and ADRs compared to single-drug therapies that are more common in other chronic diseases [12]. The incidence of ADRs varies from region to region, with India reporting 31% [30], Malaysia 42.2% [31], South Africa 44.5% [32], Nigeria 72% [32], and Ethiopia 22.9% [32]. Most of the ADR cases occurred within six months after initiation of ART [33]. 

The most prevalent cause domain was patient-related, specifically associated with medication intake adherence. A study showed that 70% of non-adherence patients developed HIV treatment failure [34]. Several studies also demonstrated that adherence was associated with HIV treatment failure [34,35]. Adherence to ART is a critical factor in HIV management. A good adherence level, which is >95%, is required to ensure treatment effectiveness and prevent HIV treatment failure and drug resistance [36,37]. Inappropriate timing of ART and patients taking complementary and alternative medicines (CAM) and illicit drugs can also contribute to the patient-related domain. Some ARV medications should be taken with or without meals for better absorption or to avoid side effects such as efavirenz [38]. The central nervous system (CNS) side effects may be due to patients failing to follow the medication intake instruction and may experience CNS side effects including nightmares or vivid dreams, dizziness, headaches, and insomnia [39]. The prevalence of illicit drug use among HIV patients was 26.69% [38]. Illicit drugs may affect the patient’s adherence to the ART [40,41] and coadministration with the ART results in toxicity [41,42]. Alcohol consumption may also influence the patients' adherence to ART [43]. Cognitive impairment that results from alcohol consumption could possibly increase the probability of non-adherence to ART [43]. A study showed that the use of CAM among HIV patients was around three times higher than among non-HIV patients [44]. A previous study has reported that the use of CAM among HIV patients in Malaysia was about 78.2% [45]. The CAM may be used to manage the ART side effects, strengthen the immune system, and enhance general well-being [46]. There are significant interactions of some ARVs with herbals such as St. John’s wort, garlic, and red rice yeast, which should be avoided [41,47].

The second most frequent cause of DRPs was categorized under other domains, such as inappropriate monitoring and ADR with no obvious cause. The monitoring of patients on ART holds a crucial role in ensuring the efficacy of treatment and identifying issues related to ART adherence [48]. Basic laboratory investigations such as HIV RNA viral load and CD4 T-lymphocyte count were required at entry to care [17]. HIV RNA viral load monitoring is required four to six months after ART initiation and six to twelve months after achieving virological suppression for more than one year [17]. CD4 T-lymphocyte count is monitored four to six months after the initiation of ARV to assess the immunological response to ART and to assess the need to discontinue opportunistic infection prophylaxis [17]. This study encountered that some laboratory monitoring, such as CD4 T-lymphocyte count and HIV RNA viral load, was not performed as suggested by guidelines because patients were considered stable by physicians. Two-thirds of patients experienced ADR after ART was initiated, and the majority were in the treatment failure group. Several studies also identified that ADR was associated with HIV treatment failure [49-51]. In the present study, the high occurrence of DRPs related to treatment safety might be due to the use of NNRTI-based regimens. More than half of the patients who started on NNRTI-based regimens experienced regimen changes. Despite a low genetic barrier to resistance with single mutations [52] and low tolerance [53], NNRTI-based regimens remain widely used as initial regimens in low- and middle-income countries, including Malaysia [54].

Another important cause of DRPs was associated with drug selection. Initiating ARV may also require special consideration; for example, patients with a baseline HIV RNA viral load >100 000 copies/mL should not start with rilpivirine-based regimens [55]. The regimens of abacavir/lamivudine with efavirenz or atazanavir/lopinavir also should be avoided [56,57]. These regimens have been observed to have a high rate of virological failure [57-59]. Efavirenz is also not recommended in patients with underlying depression as it causes CNS side effects, including suicidal ideation [17].

The present study showed a significant difference in DRP cause distribution for patient-related and monitoring needs between the treatment failure group and the treatment success group. Patient-related causes accounted for the majority of the DRPs, namely adherence level and patients unable to understand instructions properly. This described the need for enhancing patients’ knowledge and understanding of ART, followed by an evaluation approach. The patient’s education would be beneficial with the involvement of the patient’s spouse, family member, or third person who already knew about the patient’s status [60,61].

This study has identified potential problems and causes that relate to HIV treatment failure, which may enhance future strategies in HIV management. Strategies towards HIV treatment success should be targeted to the most identified causes. Although a single data collector helps to maintain consistency in data collection procedures and reduces interpretation variability, it may also introduce bias and underestimation or overestimation of DRPs. However, the study may also have some other limitations. The nature of the research design was retrospective and may be exposed to potential data omissions or misinterpretations. Baseline CD4 and duration of ART for both study groups were significantly different, which might have influenced the final outcomes of DRPs. 

## Conclusions

The occurrence of DRPs in HIV treatment failure was high. Treatment effectiveness and treatment safety were the dominant domain of problems, while patient-related factors were the common cause domain. This study addresses the challenges in HIV patient management and requires a multi-faceted approach. Healthcare providers, including physicians, pharmacists, and nurses, need to ensure that patients understand the treatment regimen, monitor closely for ART side effects, and identify barriers to ART adherence, which can help to prevent HIV treatment failure.
